# Anatomical Resections for Pulmonary Metastases: A Narrative Review of Indications, Techniques, and Outcomes

**DOI:** 10.3390/cancers17233734

**Published:** 2025-11-22

**Authors:** Alessio Campisi, Andrea Dell´Amore

**Affiliations:** 1 Department of Thoracic Surgery, Thoraxklinik at University of Heidelberg, 69126 Heidelberg, Germany; 2 Translational Lung Research Center Heidelberg (TLRC-H), German Center for Lung Research (DZL), 69126 Heidelberg, Germany; 3 Thoracic Surgery Unit, Department of Cardiac, Thoracic, Vascular Sciences and Public Health, University of Padova, 35128 Padova, Italy; andrea.dellamore@unipd.it

**Keywords:** pulmonary metastasectomy, anatomical lung resection, segmentectomy, lobectomy, minimally invasive thoracic surgery

## Abstract

**Background:** Pulmonary metastases occur in approximately 20–40% of patients with solid malignancies, with colorectal cancer representing the most frequent primary source. Surgical resection remains a potentially curative strategy for selected patients, and wedge metastasectomy has long been considered the standard of care. However, increasing attention has been paid to the role of anatomical resections such as segmentectomy and lobectomy, particularly for centrally located or technically challenging metastases. **Methods:** We performed a narrative literature review across PubMed, Embase, and Scopus databases covering the period 2010–2025, using the keywords “pulmonary metastasectomy”, “anatomical resection”, “segmentectomy”, “lobectomy”, and “pneumonectomy”. Articles included original series, comparative studies, meta-analyses, and systematic reviews. Special attention was given to technical indications, oncological outcomes, minimally invasive techniques, histological differences, and postoperative functional results. **Results:** Evidence suggests that anatomical resections are most often indicated not purely on oncological grounds, but due to technical considerations such as central localization, size greater than 2 cm, involvement of segmental or lobar bronchi or vessels, or the presence of multiple metastases confined to one lobe. Comparative studies indicate that segmentectomy provides superior local control compared to wedge resection, while lobectomy ensures complete clearance in complex cases, with five-year overall survival approaching 50% in carefully selected colorectal cancer patients. **Conclusions:** While wedge resection and metastasectomies remain the gold standard for small, peripheral metastases, anatomical resections represent a valuable extension of the surgical armamentarium in secondary lung cancer. Their role should be understood primarily as a technical necessity to ensure radical clearance while minimizing loss of lung parenchyma. The integration of minimally invasive approaches and emerging adjuncts, such as 3D reconstruction and fluorescence-guided surgery, is likely to further refine patient selection and optimize outcomes.

## Simple Summary

Pulmonary metastases represent one of the most frequent manifestations of systemic malignancies. Wedge resection has long been the surgical mainstay; however, anatomical resections such as segmentectomy and lobectomy are gaining prominence in complex cases. This narrative review critically evaluates the evidence for anatomical resections in secondary lung cancer, analyzing technical indications, patient selection, histological nuances, functional outcomes, and oncological benefits. We emphasize that these procedures are often driven more by technical necessity, such as deep, central, or large lesions, than by intrinsic oncological superiority, though select data suggest improved local control. The integration of minimally invasive approaches, particularly video- and robotic-assisted thoracic surgery, supports broader application of anatomical resections. Future directions include the use of 3D reconstruction, fluorescence-guided surgery, and molecular profiling to refine indications and outcomes.

## 1. Introduction

Pulmonary metastases represent one of the most frequent manifestations of systemic malignancies [1]. They occur in up to 40% of patients with solid tumors and are particularly common in colorectal cancer, renal cell carcinoma, sarcomas, and breast cancer [1,2]. The biological rationale for surgical treatment rests upon the principle of oligometastatic disease, a state in which a limited number of secondary lesions can be treated with curative intent, potentially prolonging survival and, in some cases, offering long-term disease control [3,4]. Since the landmark series of Thomford in the 1960s [5], pulmonary metastasectomy has been progressively incorporated into oncological practice, evolving from a controversial procedure into a widely accepted strategy for carefully selected patients.

The surgical management of pulmonary metastases has historically favored wedge resections. This approach is justified by its parenchymal-sparing nature, its technical feasibility, and the fact that metastases are often peripheral and small. Over the past decades, wedge metastasectomy has been considered the standard, and large retrospective series have confirmed its safety and efficacy in terms of survival [1,6]. Nonetheless, wedge resections have limitations, particularly when lesions are located deep in the parenchyma, in close proximity to major vessels or bronchi, or when multiple lesions occupy the same lobe. In such scenarios, anatomical resections, segmentectomy, lobectomy or even more complex procedures, become necessary to achieve complete resection with adequate margins [7].

The distinction between oncological and technical indications is of paramount importance. Unlike primary lung cancer, where anatomical resections have a clear oncological rationale, the benefit of anatomical resections in metastatic disease is less straightforward. Instead, these procedures are often driven by the need to ensure complete removal of the metastasis while preserving the maximum amount of healthy lung parenchyma. Segmentectomy in particular has gained traction as an intermediate approach, balancing oncological thoroughness with functional preservation. Lobectomy, while more extensive, remains the gold standard for centrally located or bulky metastases. Pneumonectomy is rarely indicated and should be reserved for exceptional cases, given its significant morbidity and impact on postoperative quality of life.

In parallel, the surgical landscape has been reshaped by the advent of minimally invasive approaches. Video-assisted thoracic surgery (VATS) and robotic-assisted thoracic surgery (RATS) have increasingly replaced open thoracotomy [7]. These techniques offer reduced postoperative pain, shorter hospital stays, and faster functional recovery, without compromising oncological outcomes [8]. In the setting of metastasectomy, where patient selection is crucial and many individuals may undergo repeated thoracic procedures, the benefits of minimally invasive surgery are particularly relevant. However, an important limitation of VATS and RATS in the context of pulmonary metastasectomy is the absence of tactile feedback. In open surgery, manual palpation of the lung allows the surgeon to detect occult metastases not visible on preoperative imaging. The inability to palpate may lead to missed lesions during minimally invasive procedures [9,10,11,12]. This limitation may be evaluated with the development of preoperative Three-dimensional (3D) reconstruction, intraoperative ultrasound, fluorescence-guided surgery, and tactile sensor systems to improve nodule localization.

The current review aims to provide an integrative narrative synthesis of the available literature regarding anatomical resections in secondary lung cancer. We will examine the indications, techniques, and perioperative management of segmentectomy, lobectomy, and pneumonectomy for pulmonary metastases, with a focus on technical challenges, minimally invasive surgery, histological subtypes, functional outcomes, and long-term oncological results. Moreover, we will critically assess the comparative role of wedge versus anatomical resections, with special emphasis on whether the latter should be considered oncologically superior or primarily a technical necessity.

## 2. Materials and Methods

This work represents a narrative review aimed at summarizing and critically interpreting the available evidence on anatomical resections for pulmonary metastases. A comprehensive literature search was performed using the PubMed, Embase, and Scopus databases, covering the period from January 2010 to March 2025. The search combined medical subject headings (MeSH) and free-text terms including “pulmonary metastasectomy”, “anatomical resection”, “segmentectomy”, “lobectomy”, “pneumonectomy”, and “secondary lung cancer”. The selection of articles was purposeful rather than systematic, focusing on studies that best illustrate the indications, techniques, and outcomes of anatomical resections. Both original series and comparative studies were considered, as well as systematic reviews, meta-analyses, and expert consensus papers published in peer-reviewed journals. Relevant references cited in these articles were also reviewed to identify additional pertinent works.

Throughout this review, the term ‘secondary lung cancer’ is used to describe pulmonary metastases originating from extrapulmonary primary malignancies. It should not be interpreted as a new or independent primary lung tumor, in order to avoid terminological ambiguity.

The review pays particular attention to comparative analyses of wedge versus anatomical resections, the impact of minimally invasive approaches (VATS and RATS), histology-specific outcomes, and functional consequences of different resection types. Recent systematic reviews were prioritized for their comprehensive synthesis of existing evidence, alongside landmark retrospective series and population-based analyses.

## 3. Indications for Anatomical Resections

The rationale for anatomical resections in pulmonary metastasectomy differs significantly from that in primary lung cancer. While in non-small cell lung cancer, anatomical resections remain the oncological gold standard due to lymphatic spread along bronchovascular structures [13,14,15], in metastatic disease, the primary goal is radical excision of the metastatic deposit with adequate margins while sparing lung parenchyma. Thus, the indications for segmentectomy, lobectomy, or pneumonectomy are predominantly technical rather than oncological.

Several scenarios mandate anatomical resections as shown in Table 1.

Central location of metastases adjacent to major bronchi or pulmonary vessels is a frequent indication, as wedge resection in such cases would risk incomplete excision or inadequate margins [4]. Similarly, deep-seated metastases in segmental parenchyma often require segmentectomy to achieve sufficient clearance [6]. Lesions larger than 2 cm or those abutting segmental or lobar bronchi are also best managed by anatomical resection to achieve adequate margins and prevent incomplete excision. In addition, non-anatomical resection of larger or deeply located metastases may cause distortion of adjacent bronchi or segmental vessels, leading to partial atelectasis or non-functional parenchyma that would not be effectively preserved by wedge resection. Anatomical resection in such cases ensures the removal of compromised tissue and reduces the risk of postoperative infection or residual non-ventilated lung segments. Another relevant indication is the presence of multiple metastases confined to a single lobe, where lobectomy may ensure complete clearance with fewer parenchymal divisions compared to multiple wedge resections [7].

Lymph node involvement is a debated indication. While the prognostic impact of mediastinal lymph node metastases is uniformly poor, hilar or intrapulmonary nodal disease does not invariably preclude surgery. In such cases, anatomical resections combined with formal lymphadenectomy may provide both staging accuracy and potential oncological benefit [16,17].

Importantly, indications for pneumonectomy are exceedingly rare. Pneumonectomy for metastases is associated with high morbidity and mortality and should be reserved for highly selected patients where complete resection is otherwise impossible [18].

Thus, while wedge metastasectomy remains the standard for peripheral and small lesions, anatomical resections are indispensable in scenarios where technical factors dictate the need for more extensive surgery.

## 4. Surgical Techniques

### 4.1. Segmentectomy

Segmentectomy represents the most lung-preserving form of anatomical resection, making it especially suitable for deep-seated metastases that cannot be adequately treated with wedge resection. It is also a preferred option for patients with compromised pulmonary function, as it maximizes parenchymal conservation while maintaining oncological adequacy [13,14].

In comparative studies, segmentectomy has demonstrated lower rates of local recurrence compared to wedge resection. Shiono et al. [6], in a cohort of 128 patients, found that segmentectomy achieved 55% five-year overall survival with only 8% local recurrence, compared to significantly higher recurrence rates after wedge resections. Functional preservation after segmentectomy is superior to lobectomy, with postoperative declines in FEV_1_ generally less than 10%, compared to 15–20% following lobectomy [19,20]. These findings confirm the parenchyma-sparing advantage of segmentectomy while maintaining adequate oncological margins. Similarly, the postoperative DLCO reduction is significantly lower after segmentectomy, favoring better long-term pulmonary function and tolerance to repeat resections.

### 4.2. Lobectomy

Lobectomy remains the most frequent anatomical resection in metastasectomy [7]. It is the procedure of choice when metastases are centrally located within a lobe, when multiple nodules occupy the same lobe, or when there is involvement of lobar bronchi or vessels. Lobectomy provides a radical clearance while preserving uninvolved lobes, thus balancing oncological thoroughness and functional preservation.

Prisciandaro et al. [7] performed an exploratory systematic review and concluded that lobectomy can be justified in selected patients, particularly those with colorectal cancer metastases, where five-year overall survival reached 50% in large series. Importantly, perioperative morbidity of lobectomy in the metastatic setting does not appear significantly higher than in primary lung cancer surgery, especially when performed via minimally invasive approaches.

### 4.3. Pneumonectomy

Pneumonectomy is rarely indicated for pulmonary metastases. When required, it is usually due to massive hilar involvement or multiple lesions spanning across lobes within one lung. The morbidity and mortality associated with pneumonectomy are high, with significant long-term impact on quality of life. It is emphasized that pneumonectomy should be avoided whenever possible, and that alternative strategies such as combined resections or staged metastasectomies should be considered [21].

### 4.4. Minimally Invasive Approaches

The widespread adoption of VATS and RATS has significantly changed the surgical landscape. Multiple retrospective series and meta-analyses confirm that VATS metastasectomy provides equivalent oncological outcomes compared to open thoracotomy, with the added benefits of reduced postoperative pain, shorter hospital stay, and faster return to normal activities (Table 2) [7,9,10,11,12]. Robotic-assisted techniques further enhance precision in anatomical resections, facilitating fine dissection of segmental bronchi and vessels [22]. Although RATS offers enhanced dexterity and superior three-dimensional visualization, its perceived longer learning curve primarily reflects the early stages of technology adoption, including docking time and system setup, rather than intrinsic operative complexity. As experience accumulates, this difference becomes minimal, with comparable procedural times to VATS [23].

Importantly, patients undergoing pulmonary metastasectomy often require repeat interventions due to recurrence. Minimally invasive approaches are advantageous in this setting as they reduce postoperative adhesions and facilitate reoperations. Reports have underlined the safety and feasibility of repeated minimally invasive metastasectomies, supporting their role as the preferred approach in modern thoracic oncology [21,24].

## 5. Oncological Outcomes

The oncological outcomes of anatomical resections in pulmonary metastasectomy have been the focus of intense debate. While wedge resection remains the standard for peripheral and small metastases, segmentectomy and lobectomy offer improved local control in anatomically challenging cases.

Several retrospective studies have reported a survival advantage with anatomical resections. Table 3 provides an overview of the major studies comparing wedge and anatomical resections, summarizing oncological outcomes across different series.

Shiono et al. [6] demonstrated that segmentectomy significantly reduced local recurrence compared to wedge resections, particularly in deep lesions, without increasing perioperative morbidity. Similarly, Liu et al. [17] reported that anatomical resections achieved improved disease-free survival (HR 0.54, 95% CI 0.30–0.97, *p* = 0.041) in patients with colorectal lung metastases. In addition, the prospective multicenter GECMP-CCR study by Hernández et al. [18] demonstrated that major anatomical resections (lobectomy or segmentectomy) were associated with superior disease-specific survival (58% vs. 42% for limited resections) and lower local recurrence rates. Prisciandaro et al. [7] noted in their systematic review that lobectomy, when indicated, provided oncological outcomes comparable to wedge resections but with a lower risk of margin involvement. Five-year overall survival rates for lobectomy in colorectal cancer metastases range between 40% and 50%, emphasizing its validity in carefully selected patients. Taken together, these findings underline that although anatomical resections may not universally confer a survival advantage, they can enhance local disease control and staging accuracy in anatomically complex or centrally located metastases.

However, the survival benefit of anatomical resections is not universal and largely depends on patient selection, disease biology, and completeness of resection. Several studies indicate that complete resection (R0) remains the most important prognostic factor, irrespective of the type of resection [21]. Local recurrence after anatomical resections appears lower, but distant recurrence remains common, reflecting the systemic nature of metastatic disease.

Thus, the oncological role of anatomical resections lies less in extending survival universally and more in ensuring complete clearance in patients where wedge resection is insufficient due to technical reasons.

## 6. Functional Outcomes

Functional preservation is a central consideration in pulmonary metastasectomy, particularly as many patients undergo repeated resections. Anatomical resections inevitably sacrifice more lung parenchyma than wedge resections, raising concerns about postoperative pulmonary reserve.

Segmentectomy offers an excellent compromise, combining oncological security with minimal functional loss. Postoperative reductions in FEV1 and Diffusing capacity of the lung for carbon monoxide (DLCO) after segmentectomy are typically modest, significantly less than after lobectomy [19,20]. In contrast, lobectomy may lead to a 10–20% reduction in FEV1, which, although well tolerated in patients with good baseline function, may preclude further resections in case of recurrence. Pneumonectomy remains the most functionally devastating, with severe impairment in exercise tolerance and quality of life.

Minimally invasive approaches further mitigate functional decline by reducing pain, limiting chest wall trauma, and facilitating early mobilization. Enhanced recovery after surgery (ERAS) protocols have also been integrated into metastasectomy, promoting early ambulation, optimal analgesia, and shorter hospital stays, which indirectly preserve functional status [25].

## 7. Histology-Specific Considerations

The impact of primary tumor histology on the outcomes of anatomical resections is significant.

- Colorectal cancer (CRC): CRC remains the most common source of pulmonary metastases. Multiple studies, including the review by Prisciandaro et al. [7], confirm that anatomical resections provide favorable outcomes in centrally located or large CRC metastases. Five-year overall survival rates after complete resection exceed 40%, with lobectomy demonstrating comparable results to wedge resection when margins are clear.
- Sarcomas: Pulmonary metastasectomy for sarcoma is frequently repeated due to high recurrence rates. In this context, parenchymal preservation is critical, making wedge resections preferable. However, for deep or central metastases, segmentectomy may be justified.
- Renal cell carcinoma (RCC): RCC metastases often present as solitary and indolent lesions. Anatomical resections are rarely necessary, but in central locations, lobectomy can achieve durable disease control.
- Other histologies: breast cancer, melanoma, and head and neck squamous carcinoma represent less frequent sources of pulmonary metastases. In these groups, systemic therapy often plays a dominant role, and surgery is reserved for oligometastatic disease [26,27,28]. Anatomical resections are indicated only for technical necessity rather than oncological rationale.

Thus, the role of anatomical resections varies according to histology, with CRC being the setting where lobectomy and segmentectomy are most frequently justified.

## 8. Discussion

Anatomical resections, once reserved for primary lung malignancies, have now entered the therapeutic armamentarium for metastases, especially in complex or central cases. Anatomical resections such as segmentectomy and lobectomy provide several potential advantages, clearer margins, improved access for lymphadenectomy, and anatomical orientation that may reduce local recurrence [6,7]. These are particularly valuable in colorectal cancer metastases [17], where central lesions or multiple metastases in one lobe can be approached through anatomical resections to minimize residual disease [18].

The decision to perform an anatomical resection in metastatic disease is primarily driven by technical necessity rather than intrinsic oncological superiority. However, as recent data suggest [6,17,18], the boundary between these rationales is becoming increasingly blurred. Anatomical resections have been associated with improved disease-free survival and local control in selected patients, particularly those with limited, slow-growing metastases, largely because they enable complete (R0) resection and accurate staging. The oncological value of these procedures is therefore indirect but clinically meaningful, reflecting their capacity to deliver radical clearance rather than a distinct biological effect.

Importantly, several studies have shown that the type of resection (wedge versus anatomical) does not independently influence survival when complete resection is achieved [21]. The biology of the disease remains the primary prognostic driver. Nonetheless, local control is a necessary condition for long-term survival, and anatomical resections often offer more secure margins in deep-seated or central lesions. Additionally, the possibility of repeat metastasectomy further supports strategies that preserve lung parenchyma, which argues in favor of segmentectomy over lobectomy where feasible. Thus, the choice between wedge, segmental, and lobar resection should be individualized, balancing radicality with parenchymal preservation. Segmentectomy, when technically feasible, provides a favorable compromise—offering oncological completeness with minimal functional impairment. The possibility of repeated metastasectomy underscores the need to preserve pulmonary reserve, supporting a parenchyma-sparing philosophy consistent with ESTS consensus recommendations [13,16].

Patient selection is paramount. Tools such as volumetric imaging, 3D reconstructions, and intraoperative indocyanine green (ICG) fluorescence are enhancing the surgeon’s ability to plan and execute precise anatomical resections [29,30]. Moreover, the integration of molecular profiling may soon allow better stratification of which patients are likely to benefit from aggressive local therapy versus those who should be prioritized for systemic treatment. Minimally invasive techniques, particularly RATS, have reduced the morbidity historically associated with anatomical resections [23,31]. Robotic platforms provide superior visualization and maneuverability in tight anatomical spaces, potentially facilitating more widespread adoption of segmentectomies in metastatic disease. Centers with high expertise are increasingly reporting excellent outcomes even for redoing anatomical resections using robotic platforms [23].

Future randomized controlled trials comparing anatomical and non-anatomical resections remain challenging due to ethical and logistical issues. However, large prospective registries, such as the PulMiCC study [32] and its successors, may shed light on the long-term role of anatomical resections. As more granular data becomes available, especially with centralized databases and molecular annotation, the thoracic oncology community will be better positioned to identify optimal candidates and tailor surgical strategies accordingly.

Finally, as the field advances, guidelines from leading societies such as the European Society of Thoracic Surgeons (ESTS) and the Society of Thoracic Surgereons (STS) will need to reflect emerging evidence on anatomical resections. Clearer definitions of indications, especially in histologies beyond colorectal cancer, will help standardize practice and ensure equitable access to advanced surgical options. In fact, despite the growing body of retrospective and registry-based data, the current evidence supporting anatomical resections in metastatic disease remains heterogeneous and largely observational. There are no level I trials directly comparing anatomical and non-anatomical resections in this setting, mainly due to ethical and logistical barriers. Consequently, most conclusions rely on institutional experience, expert consensus, and database analyses rather than randomized evidence.

Future progress will depend on the integration of international registries, standardized reporting, and multidisciplinary collaboration to refine patient selection and clarify whether anatomical resections truly confer an oncological benefit beyond technical radicality. These limitations should be recognized when interpreting the present literature and developing future guidelines.

## 9. Clinical and Biological Interpretation

The oncological value of anatomical resections in metastatic disease should be interpreted within the evolving concept of tumor biology rather than as a purely technical choice. From our perspective, anatomical resections do not intrinsically modify the natural history of metastatic cancer; instead, they optimize the conditions under which curative surgery can be achieved. By following segmental and lobar planes, surgeons can ensure wider and anatomically consistent margins, remove potential microvascular or peribronchial tumor deposits, and perform accurate lymph node sampling. These elements collectively improve local control and the quality of oncological staging.

From a biological standpoint, patients who benefit most from anatomical resections are likely those with limited tumor burden, indolent disease kinetics, and the capacity to tolerate repeated resections, features consistent with an “oligometastatic” state. In these patients, anatomical resection becomes not simply a technical refinement but a tool for durable control of locoregional disease. However, its contribution remains conditional on achieving complete resection (R0) and on patient selection guided by tumor biology rather than by the size of the resection alone.

Therefore, in our opinion, the “oncological role” of anatomical resections is indirect but meaningful: they act as enablers of radicality, precision, and recurrence-prevention in anatomically or technically demanding cases, rather than as inherently superior oncological procedures.

## 10. Future Directions

The field of pulmonary metastasectomy is evolving rapidly, driven by advances in imaging, surgical technology, and tumor biology. Several key directions are likely to define the future landscape of anatomical resections in secondary lung cancer.

First, patient selection will become increasingly biology-driven. Molecular and genomic profiling of primary and metastatic tumors is expected to identify subgroups with favorable biology who may benefit from aggressive local therapies. Integrating molecular markers with radiologic and clinical criteria could refine current indications and help distinguish truly oligometastatic disease from early systemic spread.

Second, technological innovation will continue to enhance precision and safety. Three-dimensional reconstructions and virtual surgical planning are already improving preoperative mapping of segmental anatomy and resection margins. Intraoperative tools such as indocyanine green (ICG) fluorescence, navigation bronchoscopy, and tactile sensor systems may overcome the current limitations of minimally invasive techniques, particularly the loss of manual palpation. These tools will allow better detection of occult lesions and more accurate delineation of intersegmental planes.

Third, the expansion of minimally invasive and RATS is anticipated to redefine the standard of care for anatomical resections. As robotic systems become more accessible and refined, complex segmentectomies and even repeat resections will increasingly be performed with reduced morbidity. Comparative, prospective studies are needed to confirm the long-term oncological equivalence of these approaches to open surgery.

Fourth, integration of multidisciplinary and longitudinal management will become central. The decision to perform an anatomical resection should be made within a structured multidisciplinary tumor board, taking into account systemic treatment options, pulmonary function, and long-term resectability in case of recurrence. Close collaboration between thoracic surgeons, oncologists, radiologists, and molecular pathologists will optimize outcomes and resource utilization.

Finally, the creation of large, international registries and prospective cooperative trials is crucial. Current evidence remains largely retrospective and heterogeneous. Shared databases could provide high-quality real-world data to guide evidence-based surgical decision-making. These collaborative efforts will help clarify whether anatomical resections confer measurable survival advantages beyond technical feasibility and local control.

In conclusion, the future of anatomical resections in secondary lung cancer lies in precision surgery, an individualized approach combining accurate anatomical techniques, advanced imaging, and molecular insight to achieve maximal oncological benefit with minimal functional compromise.

## 11. Conclusions

Anatomical resections, including segmentectomy and lobectomy, have emerged as essential components in the surgical management of pulmonary metastases. Their indication is primarily technical rather than oncological, reserved for centrally located, deep-seated, or large lesions, or for multiple metastases confined to a single lobe. When performed in well-selected patients, these procedures ensure complete resection with satisfactory preservation of pulmonary function.

The choice between wedge resection and anatomical resection should not be viewed as a dichotomy but as a complementary continuum within a patient-centered strategy. Minimally invasive techniques, including video- and robotic-assisted surgery, have further expanded the applicability of anatomical resections, reducing perioperative morbidity while maintaining oncological efficacy.

Ultimately, anatomical resections represent a cornerstone of modern thoracic oncology surgery for secondary lung cancer. The integration of advanced imaging, perioperative optimization, and multidisciplinary evaluation will continue to refine their role, ensuring both oncological control and functional preservation.

## Figures and Tables

**Table 1:** Indications for Anatomical Resections in Pulmonary Metastases.

Indication	Preferred Resection
Central metastases near bronchi/vessel	Lobectomy
Deep-seated parenchymal lesions	Segmentectomy
Lesion > 2 cm	Segmentectomy or lobectomy
Multiple metastases in one lobe	Lobectomy
Hilar/intrapulmonary lymph node involvement	Segmentectomy/lobectomy/lymphadenectomy
Limited pulmonary reserve	Segmentectomy

**Table 2:** Comparison of Surgical Approaches for Pulmonary Metastasectomy.

Surgical Approach	Advantages	Limitations
Open thoracotomy	Manual palpation, full access to lung surface	Higher morbidity, longer recovery
VATS (Video-Assisted Thoracic Surgery)	Minimally invasive, reduced pain, shorter recovery	Limited tactile feedback, limited in complex resections
RATS (Robotic-Assisted Thoracic Surgery)	Enhanced dexterity and visualization for anatomical dissection	Cost, learning curve, lack of tactile sensation

**Table 3:** Comparative studies evaluating wedge vs. anatomical resections for pulmonary metastasectomy.

First Author (Year)	Journal	Study Type/Population	Sample (n)	Main Findings	Limitations
Shiono et al. (2017) [6]	*Eur J Cardiothorac Surg* 51(3):504–510	Retrospective, 128 pts with CRC pulmonary metastases	128	5-year OS 55% for segmentectomy vs. 45% for wedge; local recurrence 8% vs. 18% (*p* < 0.05).	Retrospective, single-center.
Liu et al. (2021) [17]	*Cancer Manag Res* 13:9429–9437	Retrospective, single-center, 94 pts	94	Anatomical resection improved DFS (HR 0.54, 95% CI 0.30–0.97, *p* = 0.041); similar OS.	Retrospective design; limited sample.
Hernández et al. (2016) [18]	*Ann Oncol* 27(5):850–855	Prospective multicenter (GECMP-CCR registry), 232 pts	232	Major resections improved DSS (58% vs. 42%) and reduced local recurrence.	Selection bias; CRC-only cohort.

## Abbreviations

3D	Three-dimensional
CRC	Colorectal Cancer
DLCO	Diffusing Capacity of the Lung for Carbon Monoxide
ERAS	Enhanced Recovery After Surgery
ESTS	European Society of Thoracic Surgeons
FEV1	Forced Expiratory Volume in 1 Second
ICG	Indocyanine Green
R0	Complete Tumor Resection with Negative Margins
RATS	Robotic-Assisted Thoracic Surgery
RCC	Renal Cell Carcinoma
STS	Society of Thoracic Surgeons
VATS	Video-Assisted Thoracic Surgery

## References

[1] Pastorino U. (1997). Lung Metastasectomy: Why, When, How. Crit. Rev. Oncol. Hematol..

[2] Pastorino U., Buyse M., Friedel G., Ginsberg R.J., Girard P., Goldstraw P., Johnston M., McCormack P., Pass H., Putnam J.B. (1997). Long-Term Results of Lung Metastasectomy: Prognostic Analyses Based on 5206 Cases. J. Thorac. Cardiovasc. Surg..

[3] Hellman S., Weichselbaum R.R. (1995). Oligometastases. J. Clin. Oncol..

[4] Treasure T., Fallowfield L., Farewell V., Ferry D., Lees B., Leonard P., Macbeth F., Utley M. (2009). Pulmonary Metastasectomy in Colorectal Cancer: Time for a Trial. Eur. J. Surg. Oncol..

[5] Thomford N.R., Woolner L.B., Clagett O.T. (1965). The Surgical Treatment of Metastatic Tumors in the Lungs. J. Thorac. Cardiovasc. Surg..

[6] Shiono S., Okumura T., Boku N., Hishida T., Ohde Y., Sakao Y., Yoshiya K., Hyodo I., Mori K., Kondo H. (2017). Outcomes of Segmentectomy and Wedge Resection for Pulmonary Metastases from Colorectal Cancer. Eur. J. Cardiothorac. Surg..

[7] Prisciandaro E., Ceulemans L.J., Van Raemdonck D.E., Decaluwé H., De Leyn P., Bertolaccini L. (2022). Impact of the Extent of Lung Resection on Postoperative Outcomes of Pulmonary Metastasectomy for Colorectal Cancer Metastases: An Exploratory Systematic Review. J. Thorac. Dis..

[8] Lim E., Harris R.A., McKeon H.E., Batchelor T.J., Dunning J., Shackcloth M., Anikin V., Naidu B., Belcher E., Loubani M. (2022). Impact of Video-Assisted Thoracoscopic Lobectomy versus Open Lobectomy for Lung Cancer on Recovery Assessed Using Self-Reported Physical Function: VIOLET RCT. Health Technol. Assess..

[9] Macherey S., Doerr F., Heldwein M., Hekmat K. (2016). Is Manual Palpation of the Lung Necessary in Patients Undergoing Pulmonary Metastasectomy?. Interact. CardioVasc. Thorac. Surg..

[10] Nakas A., Klimatsidas M.N., Entwisle J., Martin-Ucar A.E., Waller D.A. (2009). Video-Assisted versus Open Pulmonary Metastasectomy: The Surgeon’s Finger or the Radiologist’s Eye?. Eur. J. Cardio-Thorac. Surg..

[11] Murakawa T., Sato H., Okumura S., Nakajima J., Horio H., Ozeki Y., Asamura H., Ikeda N., Otsuka H., Matsuguma H. (2017). Thoracoscopic Surgery versus Open Surgery for Lung Metastases of Colorectal Cancer: A Multi-Institutional Retrospective Analysis Using Propensity Score Adjustment. Eur. J. Cardio-Thorac. Surg..

[12] Gonzalez M., Brunelli A., Szanto Z., Passani S., Falcoz P.-E. (2021). Report from the European Society of Thoracic Surgeons Database 2019: Current Surgical Practice and Perioperative Outcomes of Pulmonary Metastasectomy. Eur. J. Cardio-Thorac. Surg..

[13] Saji H., Okada M., Tsuboi M., Nakajima R., Suzuki K., Aokage K., Aoki T., Okami J., Yoshino I., Ito H. (2022). Segmentectomy versus Lobectomy in Small-Sized Peripheral Non-Small-Cell Lung Cancer (JCOG0802/WJOG4607L): A Multicentre, Open-Label, Phase 3, Randomised, Controlled, Non-Inferiority Trial. Lancet.

[14] Altorki N., Wang X., Kozono D., Watt C., Landrenau R., Wigle D., Port J., Jones D.R., Conti M., Ashrafi A.S. (2023). Lobar or Sublobar Resection for Peripheral Stage IA Non–Small-Cell Lung Cancer. N. Engl. J. Med..

[15] Ginsberg R.J., Rubinstein L.V. (1995). Randomized Trial of Lobectomy versus Limited Resection for T1 N0 Non-Small Cell Lung Cancer. Ann. Thorac. Surg..

[16] Handy J.R., Bremner R.M., Crocenzi T.S., Detterbeck F.C., Fernando H.C., Fidias P.M., Firestone S., Johnstone C.A., Lanuti M., Litle V.R. (2019). Expert Consensus Document on Pulmonary Metastasectomy. Ann. Thorac. Surg..

[17] Liu T., Chang W., Wang H., Lin Q., Wei Y., Tang W., Liu Y., Chen Y., Niu Z., Jiang Y. (2021). Anatomical Resection Improves Disease-Free Survival After Lung Metastasectomy of Colorectal Cancer. Cancer Manag. Res..

[18] Hernández J., Molins L., Fibla J.J., Heras F., Embún R., Rivas J.J., Rivas J.J., Molins L., Embún R., Rivas F. (2016). Role of Major Resection in Pulmonary Metastasectomy for Colorectal Cancer in the Spanish Prospective Multicenter Study (GECMP-CCR). Ann. Oncol..

[19] Crisafulli E., Micheletto C., Campisi A., Vocale E., Schiena C., Sartori G., Gaburro G., Felici E., Infante M. (2025). Postoperative Lung Function Advantages in Pulmonary Segmentectomy for Early--Stage Lung Cancer. Thorac. Cancer.

[20] Chen L., Gu Z., Lin B., Wang W., Xu N., Liu Y., Ji C., Fang W. (2021). Pulmonary Function Changes after Thoracoscopic Lobectomy versus Intentional Thoracoscopic Segmentectomy for Early-Stage Non-Small Cell Lung Cancer. Transl. Lung Cancer Res..

[21] Mangiameli G., Cioffi U., Alloisio M., Testori A. (2022). Lung Metastases: Current Surgical Indications and New Perspectives. Front. Surg..

[22] Yamashita S., Yoshida Y., Iwasaki A. (2016). Robotic Surgery for Thoracic Disease. Ann. Thorac. Cardiovasc. Surg..

[23] Ma J., Li X., Zhao S., Wang J., Zhang W., Sun G. (2021). Robot-Assisted Thoracic Surgery versus Video-Assisted Thoracic Surgery for Lung Lobectomy or Segmentectomy in Patients with Non-Small Cell Lung Cancer: A Meta-Analysis. BMC Cancer.

[24] Carballo M., Maish M.S., Jaroszewski D.E., Yetasook A., Bauer K., Cameron R.B., Carmack Holmes E. (2009). Video-Assisted Thoracic Surgery (VATS) for Resection of Metastatic Adenocarcinoma as an Acceptable Alternative. Surg Endosc.

[25] Nelson D.B., Mehran R.J., Mena G.E., Hofstetter W.L., Vaporciyan A.A., Antonoff M.B., Rice D.C. (2023). Enhanced Recovery after Surgery Improves Postdischarge Recovery after Pulmonary Lobectomy. J. Thorac. Cardiovasc. Surg..

[26] Moonsamy P., Hompe E., Boland G.M. (2025). Pulmonary Metastasectomy for Melanoma. Thorac. Surg. Clin..

[27] Nesbit E.G., Donnelly E.D., Strauss J.B. (2021). Treatment Strategies for Oligometastatic Breast Cancer. Curr. Treat. Options Oncol..

[28] Bradley P., Montenegro C., Piazza C. (2025). Modern Management of Distant Metastases from Head and Neck Squamous Cell Carcinoma. Curr. Opin. Otolaryngol. Head Neck Surg..

[29] Le Moal J., Peillon C., Dacher J.-N., Baste J.-M. (2018). Three-Dimensional Computed Tomography Reconstruction for Operative Planning in Robotic Segmentectomy: A Pilot Study. J. Thorac. Dis..

[30] Yotsukura M., Okubo Y., Yoshida Y., Nakagawa K., Watanabe S. (2021). Indocyanine Green Imaging for Pulmonary Segmentectomy. JTCVS Tech..

[31] Zhou N., Corsini E.M., Antonoff M.B., Hofstetter W.L., Mehran R.J., Rajaram R., Roth J.A., Sepesi B., Swisher S.G., Vaporciyan A.A. (2022). Robotic Surgery and Anatomic Segmentectomy: An Analysis of Trends, Patient Selection, and Outcomes. Ann. Thorac. Surg..

[32] Treasure T., Farewell V., Macbeth F., Batchelor T., Milosevic M., King J., Zheng Y., Leonard P., Williams N.R., Brew--Graves C. (2021). The Pulmonary Metastasectomy in Colorectal Cancer (PulMiCC) Burden of Care Study: Analysis of Local Treatments for Lung Metastases and Systemic Chemotherapy in 220 Patients in the PulMiCC Cohort. Color. Dis..

